# Curability of Multiple Myeloma

**DOI:** 10.1155/2012/916479

**Published:** 2012-05-23

**Authors:** Raymond Alexanian, Kay Delasalle, Michael Wang, Sheeba Thomas, Donna Weber

**Affiliations:** University of Texas MD Anderson Cancer Center, Houston, TX 77030-4009, USA

## Abstract

Among 792 patients with multiple myeloma treated from 1987 to 2010 and assessed after 18 months, there were 167 patients with complete remission. For those 60 patients treated between 1987–1998 and with long followup, the latest relapse occurred after 11.8 years, so that 13 patients have remained in sustained complete remission for longer than 12 years (range 12–22 years). These results suggest that 3% of all patients treated during that period may be cured of multiple myeloma. In addition to immunofixation, more sensitive techniques for the detection of residual disease should be applied more consistently in patients with apparent complete remission in order to identify those with potential cure.

## 1. Introduction

In recent years, there have been major advances in the treatment of multiple myeloma, due to new agents, superior drug combinations, and widespread use of intensive therapy supported by autologous stem cells [1–5]. Thus, remission of disease has been achieved in 85–90% of currently treated patients, including 30–40% with complete remission (CR) [5–8]. This paper assessed the potential for curability among 792 patients with newly diagnosed myeloma treated at a single center over a long time span.

## 2. Patients and Methods

Between 1987–2010, we identified 792 newly diagnosed patients treated with primary, intermittent, high-dose dexamethasone-based regimens in sequential protocols (e.g., VAD, combinations with thalidomide, bortezomib, etc.) [1–3, 7, 8]. Patients older than 65 were excluded in order to assess results among those more likely to receive intensive therapy. Patients with nonsecretory or “hyposecretory” disease (e.g., only Bence Jones protein <50 mg/day) were excluded in order to define remission status clearly (Table 1). In order to assess the impact of improved treatments and the impact of prolonged complete remission (CR), we assessed outcomes separately for those treated initially between 1987–1998 or 1999–2010. None of the patients treated between 1987–1998 had received thalidomide, lenalidomide, or bortezomib at diagnosis, but approximately 25% had received at least one of these drugs upon relapse; in contrast, all of the 330 patients treated between 1999–2010 had received at least one of those drugs as part of primary or salvage therapy. Cytogenetics was not evaluated since few patients were studied during the early treatment period.

Among all patients, intensive therapy (HDT) supported by autologous stem cells had been given within 1 year to 35% of those treated between 1987–1998 and to 82% of those treated later (*P* < .01). Details of these treatments have been described [9, 10]. All patients who received the different primary therapies were combined in the analysis, as were all patients who received the different HDT. (Most patients who had not received HDT had been denied insurance coverage, especially prior to 2000). Written permission for this retrospective review was provided by our Institutional Review Board, in accordance with an assurance approved by the Department of Health and Human Services.

## 3. Clinical Response and Statistical Methods

Partial response (PR) was defined as reduction of serum myeloma protein by >50% and of Bence Jones protein by >90%. CR required disappearance of myeloma protein by immunofixation for at least 2 months [11]. Duration of CR was calculated from onset of CR to earliest sign of relapse, such as myeloma protein recurrence by immunofixation, new bone lesions, or marrow plasmacytosis. 

Survival was calculated using the Kaplan and Meier method and differences between groups compared using the log-rank test [12, 13]. Since we desired to compare survival for patients with different degrees of response, landmark analysis was conducted after 18 months to avoid the potential bias of guaranteed survival. For 12 patients with onsets of PR or CR to later rescue treatments, survival and remission were censored at such change in status [14]; survival and remission were also censored at the last electrophoresis or immunofixation for 6 patients with CR or PR who died of unrelated diseases. Thus, 131 patients who died within 18 months were excluded from the survival analysis, including 80 patients with NR, 48 patients with PR, and 3 patients with CR; 42 of the 131 patients had received HDT with treatment-related deaths in 13 patients.

## 4. Results

### 4.1. Remission and Survival

**Figure 1:**
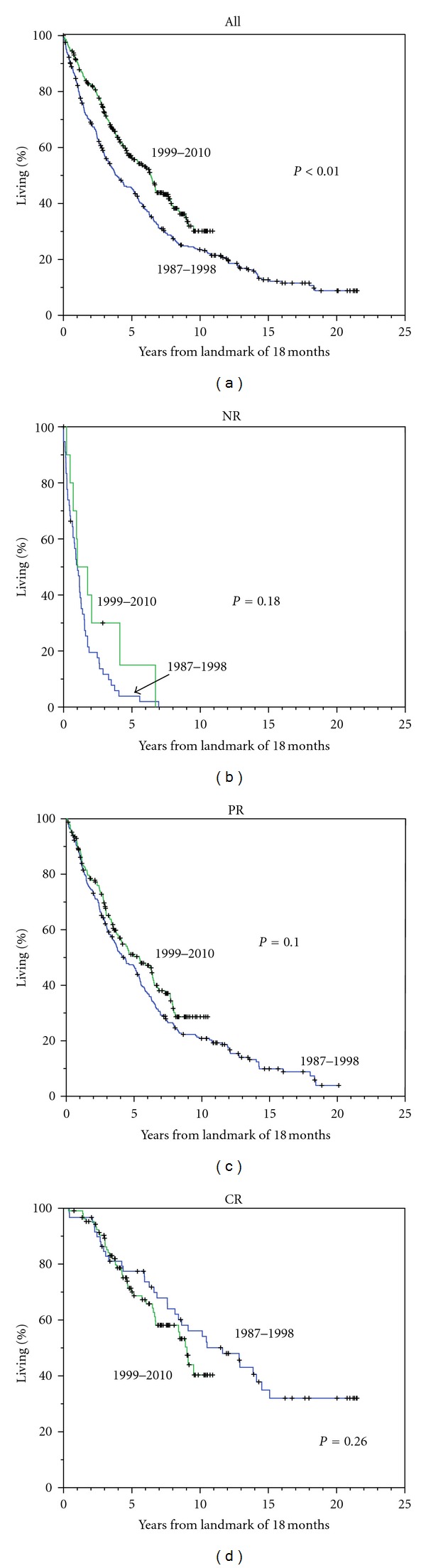
Survival after landmark of 18 months for all patients (a) and for those with NR, PR, or CR as best response at 18 months, with patients separated by early versus later treatment period. Note: longer survival among all patients treated recently but similar survival for patients with the same response status.

The best remission status at 18 months following primary treatment was defined for all 661 patients alive at that time and subsequent survival by landmark analysis was assessed for each group based on response status. Among recently treated patients, there were significantly higher frequencies of CR (36 versus 16%) and lower frequencies of NR (5 versus 16%), in comparison with earlier patients (Table 1).  Figure 1 not only depicts the longer survival for all patients treated since 1999 in comparison with the earlier group, but also the similar survival for each response status among those treated in the different time frames.

### 4.2. Complete Remission

**Figure 2:**
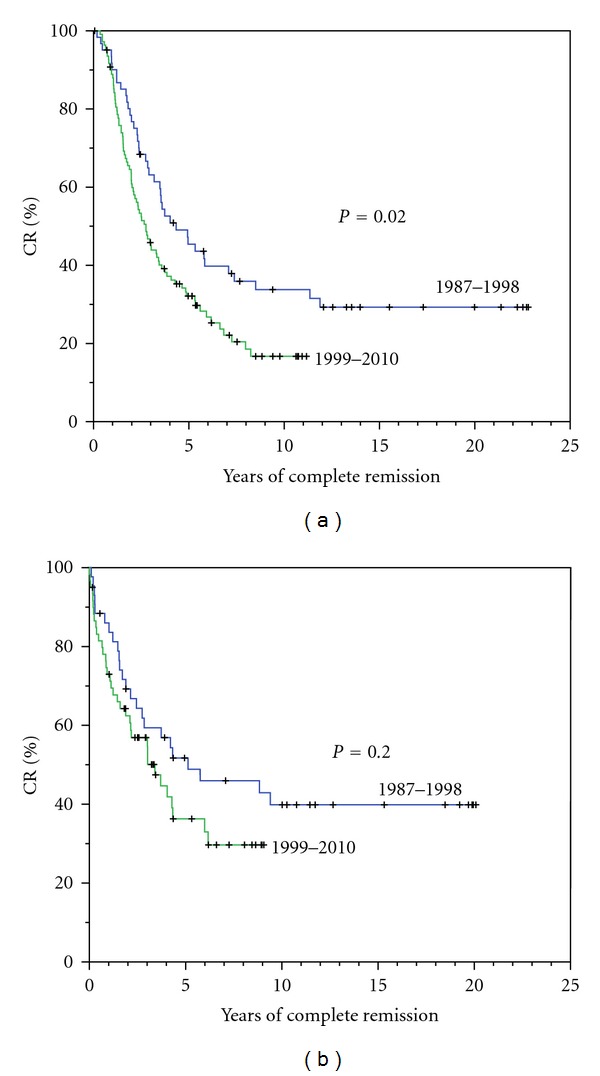
Durations of complete remission for 60 patients treated from 1987 to 1998 and for 107 patients treated from 1999 to 2010 (a). Durations of complete remission for the same group of patients after landmark of 3 years following primary therapy (b).

We focused on long-term outcomes for the 167 patients with CR at 18 months among whom 86% had received HDT, in comparison with 51% of those with NR or PR (*P* < .01).  Figure 2 shows the duration of CR for 60 patients treated between 1987–1998 and for 107 patients treated between 1999–2010, with longer duration among those treated during the earlier period (a); after landmark of 3 years following primary treatment, the differences were not significant (b). For the early treatment period, relapse of myeloma has not been seen in any patient after 12 years of CR, identifying 13 patients with sustained CR > 12 years (3% of all patients and 22% of those with CR). Clear plateaus in survival (Figure 1(a)) and in duration of CR (Figure 2(a)) were evident in patients with CR > 12 years. Yet, the higher frequency of CR with recent therapies was associated with a shorter remission time. All 13 patients with prolonged CR showed levels of uninvolved IgM that were either normal at diagnosis (>40 mg/dL) (9 patients) or had recovered to this level within 1 year of CR (4 patients).

We assessed various clinical features that might distinguish the 13 patients with CR > 12 years from the remaining 47 patients with shorter CR. There were no apparent differences in age, stage of disease, the frequency of HDT, or the pathway to reach CR with or without HDT. In addition, there was no correlation of prolonged CR with pretreatment levels of serum B_2_M, LDH, and uninvolved IgA.

## 5. Discussion

In the past, criteria for improved treatments for multiple myeloma had focused on the achievement of higher response rates and longer survival. With the wider application of new drugs and intensive therapies, progressively higher frequencies of CR have been observed in recent years [6–8, 15–17]. Among all patients treated, frequencies of CR have increased from approximately 5% prior to 1987, to approximately 15% for those treated between 1987–1998, and to approximately 30% for those treated in recent year [4–8, 15–18]. Our paper focused on long-term outcomes and the potential for cure among patients with prolonged CR. 

Criteria for complete remission, based on negative immunofixation sustained for at least 2 months, have been accepted for several years [11]. Greater sensitivities for the definition of CR have also been proposed using criteria that include the disappearance of clonal plasma cells by negative phenotype, normal molecular studies, and normal PET-scanning studies [19–21]. Further studies in large numbers of patients followed for long periods are required to clarify the added value of these procedures.

There has been recent controversy concerning the prospect of cure for patients with multiple myeloma [22–24]. The differences expressed have focused on the definition of CR and the uncertainty concerning the duration of CR. In regard to the definition of CR, many centers have considered patients with “near” CR as equivalent to CR, while others have included some patients with PR after reasoning that the residual monoclonal component may represent an MGUS status that preceded multiple myeloma [6, 22–24]. Even though many patients with “near” CR or PR have long survival, there is no evidence of a plateau in survival times for such patients, such as we describe here and others have described previously for patients with CR defined by negative immunofixation [25]. Definitions of CR that are not rigorous inflate the true frequency of CR and handicap the identification of those with meaningful potential for cure. Furthermore, Lahuerta et al. and Martinez-Lopez et al. have described significant differences in survival among patients with CR, near CR, or very good PR [5, 25]. Barlogie et al. have also shown that the implications of CR may be overrated since the duration of CR may be short (i.e., <3 years) due to early relapse of a more proliferative clone [18]. 

Following the sequence of conventional primary therapy followed by HDT supported by autologous stem cells, we observed a clear plateau in duration of CR and in survival for patients with CR sustained for at least 12 years. These findings affirm those described recently by Martinez-Lopez et al. who observed that patients treated from 1989–1998 and with sustained CR for longer than 11 years may be cured [25]. In our study, the higher frequency of CR with recent therapies was associated with shorter remission time, but this difference was not evident after requiring a landmark survival of 3 years. The validity of these findings are uncertain since the 2 groups of patients with CR were not comparable in terms of their primary therapies, the frequencies of HDT, and the unknown cytogenetic profiles. Also, Barlogie et al. have associated high frequencies of CR after intensive therapy with short durations in many patients, such as observed among many of our patients treated recently [18]. However, despite the more frequent use of HDT and new agents in the more recent cohort, the durations of CR appeared to be shorter so that CR as now defined may not be as predictive of cure as had appeared previously. 

In view of the long CR in many patients and the potential for cure in some, maintenance therapy for CR remains controversial unless prognostic factors identify those who are more likely to have short CR. Until such guidelines become available, prolonged maintenance therapy may increase the likelihood of side effects, increase the cost, and promote resistance to later drug combinations upon relapse.

## 6. Conclusion

In this retrospective review of a large number of patients with multiple myeloma, we have not observed disease relapse in any patient with CR sustained for at least 12 years. For 13 such patients, who represented 3% of those treated from 1987 to 1998, there were clear plateaus in the duration of CR and in survival that were consistent with apparent cure of multiple myeloma.

## Figures and Tables

**Table 1:** Patient population (1987–2010).

No. patients	1070		
Age > 65	−246		
Non secretory or hyposecretory	−32		

No. studied	792		
	1987–1998 (%)	1999–2010 (%)	*P*
No. patients	462	330	
Deaths < 18 *mo*	96 (21)	35 (11)	<.01
No. HDT < 12 *mo*	162 (35)	272 (82)	<.01

	Response status at 18 mo (%)		
No. patients	366	295	
NR	57 (16)	$\;\begin{array}{c} 13\;(5)\\ 175\;\;(59)\\ 107\;(36) \end{array}\}$	
PR	249 (68)		<.01
CR	60 (16)		

Duration of CR	No. (% of CR)		
>3 years	43 (72)	60 (56)	
>12 years	13 (22)	n.a.	
